# Quantifying microvascular responses to local heating using optical coherence tomography: Comparison between skin sites and sex differences

**DOI:** 10.1113/EP093337

**Published:** 2025-12-10

**Authors:** Juliene G. Costa, Kristanti W. Wigati, Louise H. Naylor, Helen Jones, Robert A. McLaughlin, Daniel J. Green

**Affiliations:** ^1^ School of Human Sciences The University of Western Australia Crawley Western Australia Australia; ^2^ Medical Physiology and Biochemistry Department Faculty of Medicine Universitas Airlangga Surabaya Indonesia; ^3^ Research Institute for Sports and Exercise Science Liverpool John Moores University Liverpool UK; ^4^ Faculty of Health and Medical Sciences University of Adelaide Adelaide Australia; ^5^ Institute for Photonics and Advanced Sensing University of Adelaide Adelaide Australia; ^6^ School of Engineering The University of Western Australia Perth Australia

**Keywords:** laser Doppler, local heating, optical imaging, regional differences, sex differences, skin microvessels

## Abstract

Due to a lack of technical capacity to directly visualise and quantify microvessels in the skin, little is known regarding regional and/or sex differences. We compared diameter, velocity, flow and density at four regional sites using a novel optical coherence tomography (OCT) approach. OCT and laser Doppler flowmetry (LDF) were performed on the back, forearm, foot and thigh in 30 healthy adults (15♂ 15♀; 31 ± 6years) at rest (33°C) and after 30 min of local heating (LH; 44°C). At baseline, larger diameter, speed, flow, density and LDF flux were recorded on the back than other sites (*P *< 0.017). In response to LH, the smallest changes in OCT‐derived diameter were observed on the back (Δ12 ± 6 µm) and foot (Δ13 ± 6 µm vs. forearm 17 ± 5 µm; thigh Δ18 ± 5 µm, all *P *< 0.005 vs. foot, back). The back exhibited the smallest change in density (back Δ19 ± 7%, forearm Δ24 ± 5%, thigh Δ26 ± 6%, foot Δ26 ± 8%, *P *< 0.02 vs. back) whilst the foot exhibited the smallest changes in speed (foot Δ27 ± 14, back Δ58 ± 22, forearm Δ47 ± 17, thigh Δ48 ± 11 µm/s, *P *< 0.001 vs. foot) and flow (Δ135 ± 60, back Δ204 ± 76, forearm Δ212 ± 60, thigh Δ247 ± 51 µL/s, *P *< 0.001 vs. foot). When sites were grouped, males had larger baseline diameters (♂ 45 ± 3 vs. ♀ 42 ± 3 µm, *P* = 0.019) and flows (♂ 109 ± 20 vs. ♀ 93 ± 17 µL/s, *P* = 0.025) whilst females exhibited larger LH‐induced changes in speed in the thigh (♀ Δ53 ± 10 vs. ♂ Δ43 ± 10 µm/s, *P* = 0.014) and density in the forearm (♀ Δ26 ± 4 vs. ♂ Δ21% ± 6%, *P* = 0.006). Regional differences exist in OCT‐derived cutaneous microvascular diameter, speed, flow and density at baseline and in response to LH. Males showed larger cutaneous diameter and flow at baseline, while females exhibited larger changes in the speed and density outcomes in response to local heating.

## Abstract (highlights)

- **What is the central question of this study?**
  Do regional and/or sex‐related differences exist in the human skin microvasculature, as quantified using optical coherence tomography at rest and in response to a local heating protocol?
- **What is the main finding and its importance?**
  Regional variations were observed in microvascular diameter, flow, speed and density. The back showed greater baseline values, and the foot exhibited the smallest heating‐induced changes. Males demonstrated larger baseline diameter and flow, whereas females showed greater heating‐induced changes in speed and density. These findings suggest heterogeneity in microvascular function, underscoring the need for regional standardisation in assessments.

## Graphical abstract

Summary of the study design, metholodgy, results and conclusions.

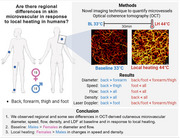

## 1 INTRODUCTION

The skin microvasculature consists of a complex and dense network of small blood vessels that play a key role in thermal and cardiovascular regulation in humans. Several techniques have traditionally been used to assess the skin microcirculation (Deegan & Wang, 2019; Low et al., 2020). Laser Doppler flowmetry (LDF) allows continuous, real‐time measurement of microvascular red blood cell movement ('flux') in arbitrary units, but these measurements are qualitative and do not directly image or quantify cutaneous microvascular blood flow. Alternative approaches, such as laser Doppler perfusion imaging and laser speckle contrast imaging, also possess limitations such as low temporal and spatial resolution (Deegan & Wang, 2019).

Cutaneous optical coherence tomography (OCT) is a novel, non‐invasive, high‐resolution imaging technique capable of providing quantitative metrics including microvessel diameter, velocity, density and flow, in vessels as small as ∼30 µm. When combined with approaches that challenge physiological function, such as skin local heating (LH), OCT can be used to assess microvascular morphology and adaptation, with enhanced between‐day reproducibility compared to that associated with laser Doppler techniques (Smith et al., 2019). Our group has previously quantified abnormal resting microvascular function in participants with heart failure (Sciarrone et al., 2022) and microvascular responses to skin heating in diabetics with and without ulcers (Argarini et al., 2020a). We have also demonstrated that OCT parameters change in response to cuff inflation (Argarini et al., 2020b), whole body heating (Carter et al., 2016) and exercise training (Argarini et al., 2021).

However, little is currently known regarding regional differences in skin microvascular morphology or physiology in humans. Some studies have suggested that distinct regions of the skin exhibit different surface temperatures, which may reflect regional variability in skin thickness, and/or vascularity (Sandby‐Møller et al., 2003). Factors such as cutaneous perfusion, along with sun exposure, have also been speculated as contributing to these differences (Sandby‐Møller et al., 2003; Waller & Maibach, 2005). In a study where LDF was used on the thigh, chest, back and forearm after mild heat stress (Inoue et al., 1998), older subjects exhibited lower flux than younger individuals, but differences between sites were not reported. In another study (Smith et al., 2013) in which forearm, abdomen, thigh and lower back skin blood flows and sweat rates were assessed in young healthy participants, higher sweat rates were evident on the back, but no differences were apparent between sites in LDF measures. Whilst these studies suggest that variations may exist in microvascular structure and function between skin sites within individuals, they were not able to visualise or quantify regional differences in parameters such as cutaneous diameter, speed, flow or density.

Sex differences in LDF measures have also been reported (Inoue et al., 2005), with higher measures in the thigh in women than men, and no differences at other sites (forehead, chest, back and forearm), raising the possibility that female reproductive hormones may modulate some aspects of the vasodilator response in different skin regions. However, sex‐ and regional differences remain unclear (Chang et al., 2023; Mustafa et al., 2022). Indeed, it was recently reported that only 34% of studies pertaining to human physiology have recruited women (Ranadive & Hagberg, 2025), emphasising the need for studies of physiological differences. The aim of the present study was to quantify microvascular OCT‐derived outcomes from distinct skin sites in response to identical local heating protocols in healthy young male and female participants. We hypothesised that differences would be apparent in diameter, speed, flow and density between anatomical sites, and that OCT responses to local heating would also be sex‐dependent.

## 2 METHODS

### 2.1 Participants

Thirty healthy adults (15 males, 15 females) aged 18–40 years were recruited. Exclusion criteria included a body mass index (BMI) greater than 30 kg/m^2^ or the use of any prescribed cardiovascular medications. In keeping with recent recommendations (Hagstrom et al., 2025), individuals reported their gender, which was consistent in all cases in this study with their sex recorded at birth. The study conformed to the standards outlined in the *Declaration of Helsinki* and was reviewed and approved by The University of Western Australia Human Research Ethics Committee (2023/ET000172). All participants provided written informed consent prior to their involvement in this study.

### 2.2 Study design

All participants attended a quiet, temperature‐controlled room (23.5 ± 1.1°C; 49.6 ± 9.8% relative humidity) in the Cardiovascular Research Laboratory at The University of Western Australia at the same time of the day (06.00–10.00 h). Participants were instructed to avoid caffeine and alcohol for 24 h prior to the test, and to refrain from prolonged sun exposure for 5 days before the testing session. If necessary, exposed hair was shaved 24 h before testing, in accordance with recommendations indicating that OCT responses to mechanical stimulation return to baseline after this period (Chaturvedi et al., 2024). The measurement sites were cleaned of cosmetics and ointments. Participants were asked to wear loose shorts and a sleeveless T‐shirt, leaving the upper part of the back accessible for scanning. At the commencement of the testing session, participant demographic data, including age, sex, menstrual cycle status and contraceptive use, were documented. After having weight and height measured to confirm their BMI, body composition was assessed using dual‐energy X‐ray absorptiometry (Lunar iDXA, GE Healthcare, Madison, WI, USA), which provided insight into fat and lean mass, and visceral adipose tissue.

### 2.3 Experimental protocol

A semi‐recumbent supine posture was maintained during the protocol, with the right arm and foot comfortably supported by foam pads to minimise movement during OCT scanning. Skin sites were prepared for LDF and OCT probe placements on the right side of the body at the following locations: the midpoint of the thigh, the proximal forearm, the upper back and the dorsum of the foot (the instep).

After initial instrumentation, local heating disks (PF450, Perimed, Stockholm, Sweden) were attached to the skin for baseline assessments at all sites, with temperature maintained at 33°C. OCT, LDF and blood pressure (Dinamap V100, GE Healthcare, Chicago, IL, USA) were collected after this 20‐min baseline period. The skin was then heated by warming the heating disks at a rate of 1°C per 10 s, from 33°C to 44°C. Upon reaching 44°C, the heater disks were maintained at 44°C for a further 30 min, when heating measurements of blood flow were collected (OCT, LDF and blood pressure).

#### 2.3.1 Optical coherence tomography

OCT is a non‐invasive high‐resolution (1–20 µm) imaging technique that can visualise and quantify vascular architecture (diameter, speed, flow and density) in human skin. The OCT imaging scanner (Telesto III, ThorLabs GmbH, Bergkirchen, Germany) captures a 3‐D scan of the skin microvasculature over a field of view of 5 × 5 × 2.5 mm (*X* × *Y* × *Z*), where the *X* and *Y* dimensions lie parallel to the skin surface, and the *Z* dimension extends into the skin. Scans were acquired at 76 kHz (A‐scans per second), giving a total scan acquisition time (at the current time) of 50 s. During scan acquisition, participants were required to remain motionless. We found this time to be well tolerated, resulting in minimal motion artefacts. A custom optical spacer was placed between the OCT scanhead and the skin to ensure an exact distance such that the focus of the OCT light beam was positioned approximately 300 µm below the skin surface to optimise the range of tissue within focus. The optical spacer also held a small heating element to gently heat the skin during the local heating protocol. The distal face of the optical spacer was covered with a small glass window to ensure a flat optical surface for scanning, and a drop of water was placed between the glass surface and the skin to reduce artefacts from mismatches in the refractive index between the glass and the skin (Liew et al., 2012).

**FIGURE 1:**
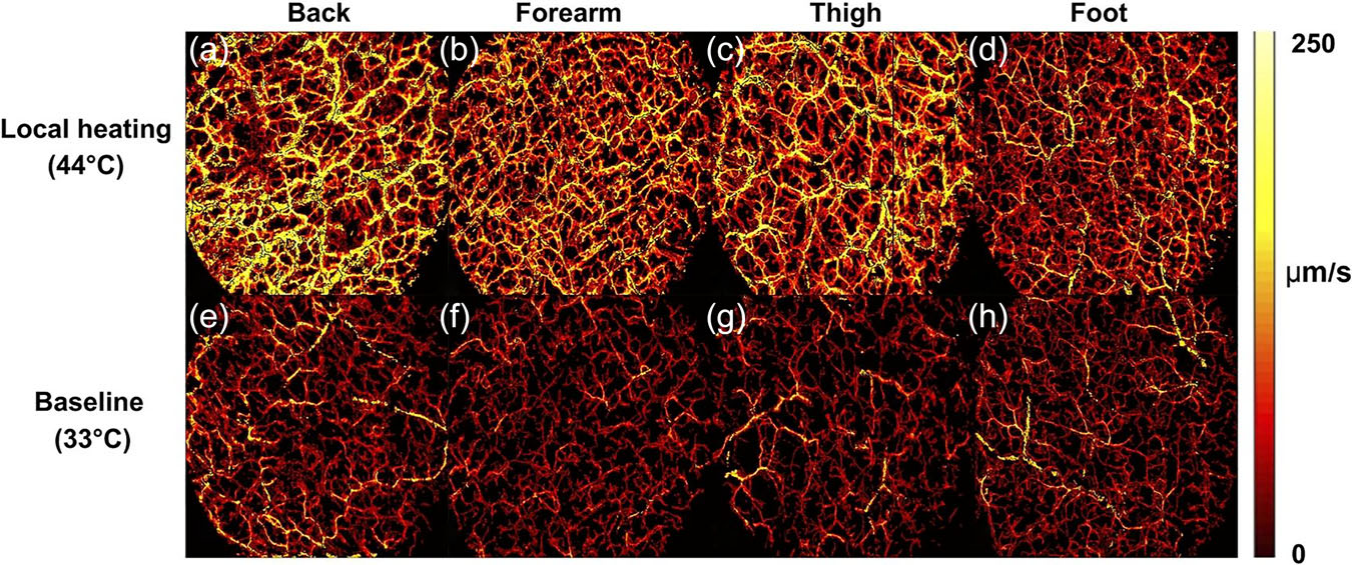
Examples of OCT‐derived images acquired at baseline (*e–h*) and after 30 min of local heating at 44°C (*a–d*) on the upper back, forearm, thigh and foot. Blood vessels are colour‐coded to indicate flow speed (µm/s).

OCT images contain a characteristic noise referred to as speckle. Although random in nature, speckle does not change over time if the tissue does not move. Blood vessels in the skin were identified by acquiring multiple A‐scans at each location during a single scan and measuring the rate of change of the speckle noise over time. Blood flow corresponded to locations where the speckle noise was measured as changing, with the rate of change related to the speed of blood flow. A 2D maximum intensity projection image (MIP) of blood flow was then generated in the *X–Y* plane, as shown in Figure 1. From these images, automated software was developed in MATLAB (MATLAB R2022b, MathWorks, Natick, MA, USA) to quantify median vessel diameter, median flow speed, median flow volume, and the vessel density for each individual scan. Vessel density was quantified as the percentage of pixels in the MIP image identified as indicating blood flow, with greater vessel density indicating greater vessel recruitment. More details of the OCT protocol have been reported by our group previously (Carter et al., 2016; Smith et al., 2019).

#### 2.3.2 Laser Doppler flux

Skin red cell flux was measured by laser Doppler flowmetry, using a 7‐Doppler array laser probe (Model 413, Periflux 5000 System, Perimed AB, Jakobsberg, Sweden). The Doppler probes were attached to the skin sites with double‐sided adhesive tape. LDF and OCT scanned the same skin area, not simultaneously. These data were exported to a data acquisition system PowerLab (LabChart 7, ADInstruments, Sydney, Australia), in real time. Skin blood red cell flux is presented in perfusion unit (PU).

### 2.4 Data analysis

The sample size calculation was based on data from the study of Hodges & Del Pozzi (2014), which observed a difference in LDF between the forearm and leg after local heating (effect size *d* = 2.06). Based on these data, a minimum of seven participants per site were required to reject the null hypothesis with 90% power and α = 0.05. G*Power software (version 3.1.9.7) was used for calculating the sample size. The Shapiro–Wilk test was used to assess the normality of the data. Blood pressure, age, height, weight, BMI, temperature and humidity, lean mass, and visceral adipose tissue passed the normality test (*P *> 0.05), and Student's *t*‐test was used to compare baseline characteristics between sexes. The Mann–Whitney *U*‐test was used to compare groups for fat mass (kg) and right leg fat (kg), as these did not pass the normality tests. Data are expressed as means ± standard deviation, unless otherwise indicated. One‐way ANOVA was performed to compare the differences between the four sites in median diameter, median flow, median speed, density and flux. A subsequent independent *t‐*test examined the differences between males and females in the change (delta, from the baseline to the local heating at each of the four sites) in median diameter. Similar analysis was performed to assess sex differences in median flow, then density, speed and flux. For all comparisons, significance was set at *P* < 0.05. Statistical analyses were performed using SPSS version 29.0 (IBM Corp., Armonk, NY, USA).

## 3 RESULTS

### 3.1 Participants characteristics

Thirty healthy adults (15 males and 15 females) participated in this study (31 ± 6 years; 28.8 kg/m^2^). Baseline characteristics are presented in Table 1. The room temperature and relative humidity were 23.5 ± 1.1°C and 49.6% ± 9.8%, with no difference in these conditions between males and females (*P* = 0.528). Females and males were well matched for age (*P* = 0.755) and BMI (*P* = 0.731). Males were taller, with higher body mass, more total and leg lean mass (kg and %), and lower percentage of fat mass. Males had higher systolic blood pressure than females, but all individuals were normotensive. No differences in heart rate or diastolic blood pressure were evident. Menstrual cycle status was self‐reported and determined using the variations described by Cole et al. (2009). Twelve women (80%) were in the luteal phase, and three (20%) were in the follicular phase of their cycle. Two (13.3%) were using an oral contraceptive, and another two (13.3%) a non‐hormonal copper intrauterine device; all four experienced regular menstrual cycles lasting 3–4 days within a 28‐day cycle.

**TABLE 1:** Participant characteristics at baseline.

	Overall (*n* = 30)	Females (*n* = 15)	Males (*n* = 15)	F vs. M *P*‐value
Room temperature (°C)	23.5 ± 1.1	23.7 ± 0.8	23.4 ± 1.4	0.528
Room humidity (%)	49.6 ± 9.8	48.9 ± 9.6	50.3 ± 10.4	0.724
Age (years)	31 ± 6	31 ± 7	31 ± 4	0.755
Height (m)	1.72 ± 0.09	1.65 ± 0.06	1.79 ± 0.05	<0.001
Weight (g)	74.3 ± 15.0	67.2 ± 14.6	81.0 ± 12.7	0.010
BMI (kg/m^2^)	24.8 ± 3.9	24.6 ± 4.5	25.1 ± 3.3	0.731
Body composition				
Lean mass (%)	69.7 ± 8.5	64.4 ± 5.6	75.0 ± 7.5	<0.001
Lean mass (kg)	52.0 ± 10.9	43.5 ± 6.4	60.4 ± 7.2	<0.001
Fat mass (%)	27.8 ± 9.3	33.8 ± 6.4	21.8 ± 7.8	<0.001
Fat mass (kg)	20.0 ± 8.7	22.5 ± 8.8	17.6 ± 8.2	0.116
Visceral adipose tissue (kg)	0.390 ± 0.405	0.228 ± 0.192	0.553 ± 0.495	0.025
Blood pressure				
SBP (mmHg)	111 ± 9	106 ± 6	115 ± 8	0.001
DBP (mmHg)	67 ± 9	64 ± 8	69 ± 10	0.074
HR (beats/min)	61 ± 8	62 ± 7	61 ± 9	0.768

*Note*: Values are means ± standard deviation. Data were compared between groups using an unpaired *t*‐test and Mann–Whitney *U*‐test for fat mass (kg) and right leg fat (kg). Statistical significance was set at *P *< 0.05. Abbreviations: BMI, body mass index; DBP, diastolic blood pressure; HR, heart rate; MAP, mean arterial pressure; RMR, resting metabolic rate; SBP, systolic blood pressure.

### 3.2 Difference between sites at baseline and in response to local heating

**FIGURE 2:**
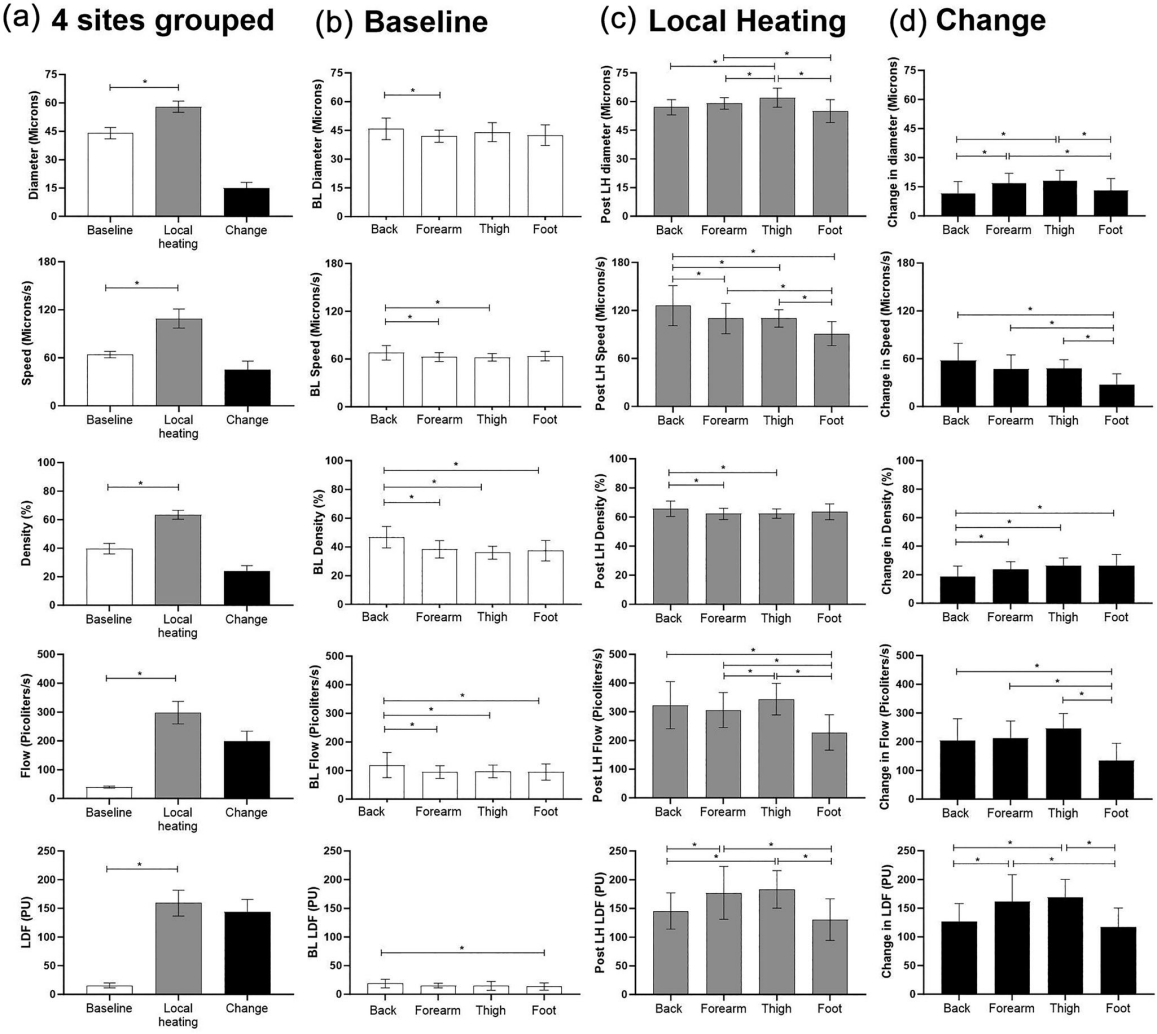
Difference between the sites at baseline, local heating and the change. Data are presented as means ± standard deviation. Data acquired from 30 participants. *Significant difference (*P *> 0.05) between sites. BL, baseline; LDF, laser Doppler flux; LH, local heating.

An example of images derived from OCT at rest and after LH on the four sites is shown in Figure 1, with quantitative results summarised in Figure 2. When all four sites were grouped (Figure 2a), there was a significant (*P *< 0.05) increase in median diameter, median speed, median flow, density and LDF (*P *< 0.001) in response to the local heating compared to baseline. When analysed as separate sites, the back (Figure 2b) exhibited a significantly larger median diameter at baseline than the forearm (*P* = 0.017) but no other sites (*P* = 0.055). Median speed was higher on the back when compared to the forearm (*P* = 0.011) and thigh (*P* = 0.006). The back exhibited higher median flow and density than the forearm (*P* = 0.012), thigh (*P* = 0.036) and foot (*P* = 0.014). The back also had a higher LDF when compared to the foot (*P* = 0.018).

The change in median diameter from baseline in response to local heating (delta value; Figure 2d) was smallest in the back, compared to the forearm (*P* = 0.002) and thigh (*P *< 0.001). The thigh had a larger change in median diameter than the foot (*P* = 0.005). The change in median speed and flow was similar on the back, forearm and thigh, and all sites exhibited higher median speed and flow than the foot (all *P *< 0.001). The change in density was similar in the forearm, foot and thigh, and all of them were higher than the back (*P* = 0.021). In LDF, the forearm and the thigh exhibited a similar increase, both larger than the back (*P* = 0.002) and the foot (*P *< 0.001).

### 3.3 Sex differences at baseline and in response to local heating

**FIGURE 3:**
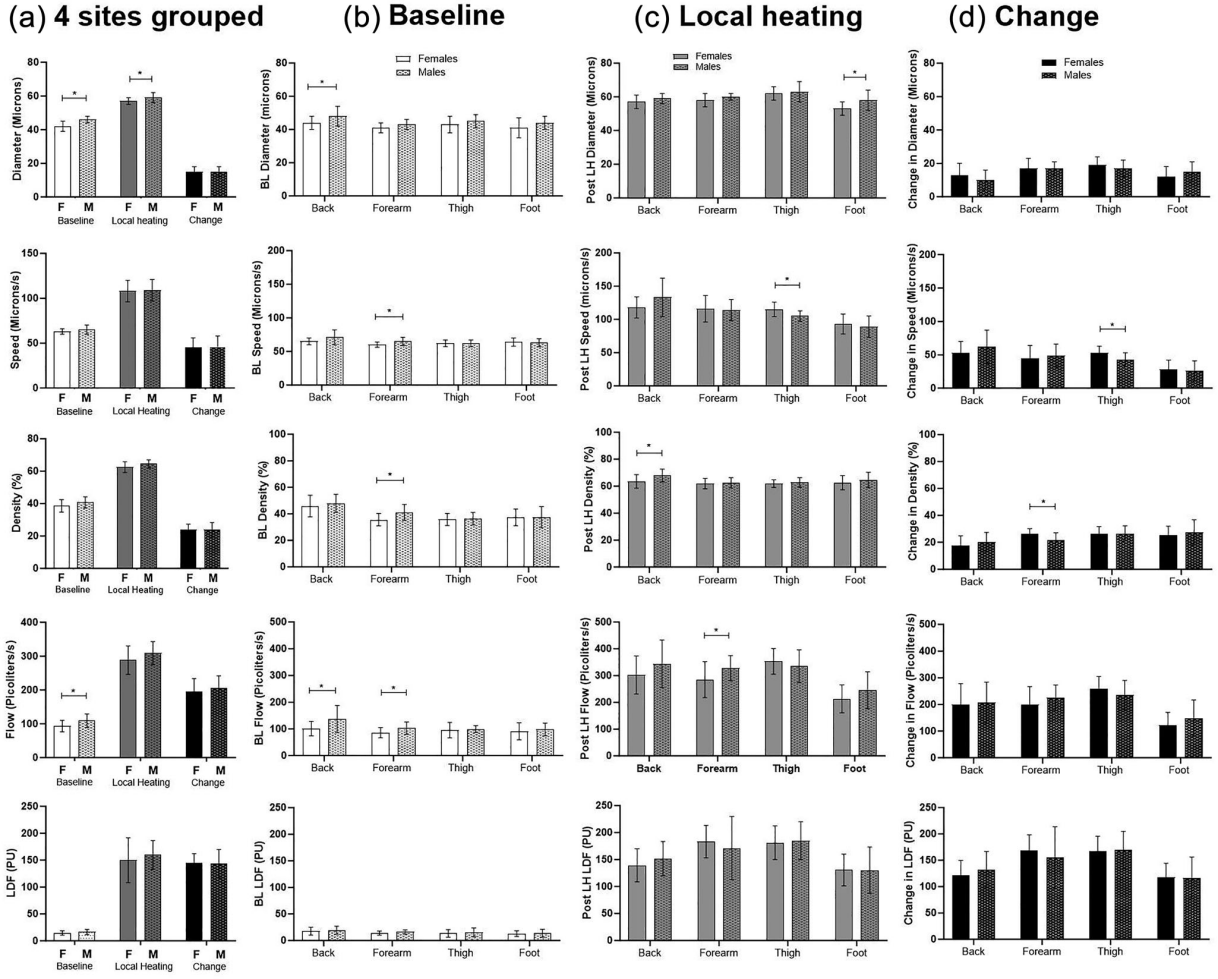
Difference between females (*n* = 15) and males (*n* = 15) at baseline, in response to local heating and the change (delta) from baseline. Data are presented as means ± standard deviation. F, females; M, males; BL, baseline; LDF, laser Doppler flux; LH, local heating. *Significant difference (*P* < 0.05) between groups.

The difference between the sexes is present in Figure 3. With four sites grouped, males exhibited larger median diameter (*P* = 0.019) and flow (*P* = 0.025) at baseline and larger median diameter in response to LH (*P* = 0.030) (Figure 3a), but with similar changes (delta from baseline) in other OCT parameters and in LDF. When the sites were analysed separately, males at baseline (Figure 3b) showed a larger median diameter on the back (*P* = 0.023), higher median speed on the forearm (*P* = 0.040), and higher median flow on the back (*P* = 0.024) and the forearm (*P* = 0.034), and higher density on the forearm (*P* = 0.008) than females. In response to local heating (Figure 3d), females had larger changes in the median speed of the thigh (*P* = 0.014) and density of the forearm (*P* = 0.006), but with no difference in median diameter and flow. No sex difference was demonstrated for LDF at baseline, or LH change.

## 4 DISCUSSION

To our knowledge, this is the first study to use an OCT approach to visualise, quantify and compare microvascular diameter, speed, flow and density across distinct regional sites under controlled ambient conditions at rest and in response to a local heating stimulus in humans. Our findings indicate that, at baseline, OCT measures derived from the back were consistently higher than those derived from other sites, while in response to local heating, OCT‐derived changes in flow were smaller in the foot than at other sites. We also observed some differences between males and females in skin responses to local heating. These findings suggest that both regional and sex differences may exist in skin microvessels in humans, with implications for previous experiments which have measured microvascular parameters at a single site.

Our findings generally suggest that LDF may be less sensitive than OCT in terms of revealing small but physiologically relevant differences between skin regions. This finding is relevant because previous studies addressing site‐specificity have relied on LDF, or even less specific plethysmographic techniques. An early study using a ‘photoelectric’ plethysmograph (Hertzman & Randall, 1948) identified higher skin blood flows on the back, compared to the foot (dorsum, plantar and toe pads) at rest, with similar patterns of flow reported in response to a heating stimulus on the forearm, trunk, leg and thigh. The authors suggested that the influence of vascular morphology in distinct regions might explain the differences they observed. In a study that employed LDF, Inoue & Shibasaki (1996) reported an increase in responses to 60 min of lower limb water immersion (42°C water bath; ambient 35°C, 45% relative humidity) that was higher in the forearm and thigh than the back and chest of young males (23 years; *n* = 10). While these findings are generally consistent with the differences we observed, our OCT findings provide insights into the diameter, velocity, density and flow parameters that are not possible using other techniques.

Our findings suggest that responses to heat stimuli may be greater in appendicular (forearm and thigh) than axial (back, chest) regions in humans. However, it was notable that both LDF and OCT responses to local heating were consistently lowest in the foot, including when compared to the thigh. The foot appeared to have less capacity to adapt to LH, with reduced flow, speed and diameter responses. Whilst the microvasculature of the foot may not contribute greatly to thermoregulation, it is clinically relevant as a common site of ulceration in diabetes and peripheral arterial disease (Bull et al., 1995; Balaz et al., 2015). Impaired microvascular reserve in the feet may relate to intrinsic anatomical factors and/or idiosyncratic microvascular architecture associated with the presence of fibrous tissue, which affords mechanical stability (Custozzo et al., 2020). We previously used OCT on the dorsum of the foot in older healthy participants with and without diabetes (Argarini et al., 2020a), and showed that diabetic individuals with ulcers exhibited larger resting diameter, velocity, flow and density measures compared to diabetic individuals without ulcers and also healthy controls, whereas diabetic individuals expressed impaired changes in response to LH. Taken together, our findings highlight the importance of comparing various skin regions to understand how ageing and pathology can impact microvascular status.

Whilst our OCT‐derived results indicating differences between appendicular and axial responses, and between those within the lower limbs (thigh vs. foot) are novel, some previous studies have compared upper and lower limb responses. Using LDF, Del Pozzi et al. (2013) reported larger contributions of nitric oxide (NO) in the calf than in the arm at rest, with no differences observed in response to LH. In another study (Hodges & Del Pozzi, 2014), spectral analyses of the LDF recordings were utilised to analyse the endothelial, sympathetic and myogenic function on the forearm and leg at thermoneutral 33°C and in response to LH (42°C). A difference between the forearm and leg was apparent in all frequencies at baseline (leg > forearm), while after LH the forearm had larger responses. The legs also exhibited a larger response to chronic exposure to haemodynamic and hydrostatic pressure gradients when upright (Pawelczyk & Levine, 2002). While these studies suggested differences between the arms and legs (excluding the feet), our OCT findings suggest similar resting and LH responses between the forearm and thigh. Future studies combining OCT and micro dialysis may provide further insights into differences in specific vasomotor pathways.

We observed some sex differences in OCT measures. Our results indicate that, when all four sites were pooled, males exhibited larger diameter and flow at baseline, due largely to higher values on the back and forearm. In response to LH, females had larger changes than males in forearm density and flow responses than those apparent in the thigh. Sex differences were also reported in a study by Cooke et al. (1990), who observed higher hand flow in males than in females at rest. However, after total body heating, the hand flow of females exceeded that of males. The authors concluded that these sex differences may be related to sympathetic vasomotor control. Sex and regional differences were also reported by Inoue et al. (2005) with changes in LDF greater on the thigh in women than men, and no differences at other sites (forehead, chest, back and forearm). Another study by Stanhewicz et al. (2014) reported that females had lower NO‐dependent vasodilation in the forearm compared to men, with no sex difference in the calf. Charkoudian et al. (1999) demonstrated that oestrogen increases cutaneous vascular responses to local warming by promoting nitric oxide synthase (NOS) activity (Hayashi et al., 1995). Sex differences in skin properties may also contribute to the above observations; males have larger epidermal thickness (Sandby‐Møller et al., 2003) and higher skin collagen (Shuster et al., 1975) and sodium content than females (Wang et al., 2017). It is unclear whether such local enhancement of vasodilation occurs uniformly across the body surface, and the inclusion of OCT outcomes, alongside modulation of sympathetic and local vasoactive pathways, may provide more detailed insight in future studies. It should also be possible to combine microdialysis and OCT techniques in future, to pharmacodissect the mechanisms responsible for regional and sex differences in cutaneous microvascular function in humans. Other studies adopting such approaches might target diseases with microvascular aetiologies, such as diabetes (Argarini et al., 2020a), heart failure (Sciarrone et al., 2022), peripheral artery disease (Kim et al., 2023) and Raynaud's phenomenon (Mustafa et al., 2022).

It is well established that oestrogen and progesterone can modify cutaneous blood flow. In our study, 80% of females were assessed during the luteal phase of the menstrual cycle. In a study by Lee et al. (2014), females in the luteal phase demonstrated greater LDF flux, skin temperature, and sweat rate compared to those in the follicular phase when exposed to a heated room (41°C, 21% RH). Similarly, Inoue et al. (2005) reported that females in the luteal phase exhibited greater skin blood flow (LDF) on the back than those in the follicular phase during heat exposure (leg heating at 42°C for 60 min) (Inoue et al., 2005). When we compared male and female results with the three females who were in the follicular phase excluded from the analysis (i.e. comparing 15♂ to 12♀ in the luteal phase), our findings remained significant and consistent with our original analyses, with larger male baseline diameters (♂ 45 ± 3 vs. ♀ 42 ± 3 µm, *P* = 0.028) and flows (♂ 109 ± 20 vs. ♀ 92 ± 19 µL/s, *P* = 0.028), and females exhibiting larger LH‐induced changes in speed in the thigh (♀ Δ53 ± 11 vs. ♂ Δ43 ± 10 µm/s, *P* = 0.022) and density in the forearm (♀ Δ27 ± 4 vs. ♂ Δ21 ± 6%, *P* = 0.008). These findings support the rationale for performing future studies that are specifically powered and designed to compare OCT parameters across distinct phases of the menstrual cycle in women.

Our study has several limitations. Despite matching age and BMI, males and females were not assessed for cardiorespiratory fitness, which might contribute to the sex differences we report. Nonetheless, we recruited healthy participants who were recreationally active but not athletes. None were taking medications. Another limitation is that race or skin type were not considered in the recruitment process. Given the known variations in skin structure and pigmentation that can influence optical and vascular properties, this should be a focus of future studies (Lee et al., 2014).

## 5 CONCLUSION

Our data support our hypothesis that regional and sex differences exist at baseline and in response to local heating. Our findings that sex‐ and site‐specific differences exist in baseline and heating‐mediated cutaneous vascular responses have implications for studies that compare individuals across clinical populations or perform within‐subject comparisons in response to interventions. This is particularly relevant when assessing the cutaneous microvasculature in diseases such as diabetes, peripheral arterial disease, and other cardiovascular diseases. OCT creates the opportunity to characterize diameter, speed, flow and density in the lower limbs, which may be particularly susceptible to early signs of atherogenesis, such as endothelial dysfunction.

## AUTHOR CONTRIBUTIONS

Juliene Goncalves Costa Dechichi: conceptualised the work, acquisition, analysis and interpretation of data for the work, writing – original draft. Kristanti W. Wigati: acquisition, methodology, analysis and interpretation of data for the work. Helen Jones and Louise H. Naylor: interpreted the data for the work, and revised the manuscript critically for important intellectual content. Robert A. McLaughlin: methodology, data curation, and revised the manuscript critically for important intellectual content. Daniel J Green: conceptualisation, supervised, data analysis, and revised the manuscript critically for important intellectual content. All authors approved the final version of the manuscript; agreed to be accountable for all aspects of the work in ensuring that questions related to the accuracy or integrity of any part of the work are appropriately investigated and resolved; and all persons designated as authors qualify for authorship, and all those who qualify for authorship are listed.

## CONFLICT OF INTEREST

R.A.M. is a co‐founder and Director of Miniprobes Pty Ltd, a company that develops optical imaging systems. Miniprobes Pty Ltd did not contribute to or participate in this study. The other authors reported no conflict of interest.

## Data Availability

The source data are available to verified researchers upon request by contacting the corresponding author.
